# Movement Notation Revisited: Syntax of the Common Morphokinetic Alphabet (CMA) System

**DOI:** 10.3389/fpsyg.2018.01416

**Published:** 2018-08-21

**Authors:** Conrad Izquierdo, M. Teresa Anguera

**Affiliations:** ^1^Faculty of Psychology, Autonomous University of Barcelona, Barcelona, Spain; ^2^Faculty of Psychology, Institute of Neurosciences, University of Barcelona, Barcelona, Spain

**Keywords:** movement behavior, observational methodology, field format, coding system, morphokinetic alphabet, grammar, movement phrase-structure, rules

## Abstract

Advances in the study of non-verbal behavior and communication have generated a need for movement transcription systems capable of incorporating continuous developments in visual and computer technology. Our research team has been working on the construction of a common morphokinetic alphabet (CMA) for the systematic observation of daily life activities. The project, which was launched several years ago, was designed to create a system for describing and analyzing body motion expression, physical activity, and physical appearance. In this paper, we describe an idiosyncratic application of Noam Chomsky’s *phrase marker grammar* to the morphokinetic phrase, the objective being to establish the grammatical rules and basic order of the symbol string according to a relational tree formed by the breakdown of the syntactic components identified as structuring the visual description of movement. Criteria for using the CMA as a coding system and a free transcription system are proposed.

## Introduction

This article discusses the theoretical and methodological aspects of the behavior stream through consideration of the problem of movement notation (Hadar, 1994). As Freedman (1981) has pointed out, the analysis of movement behavior by means of narrative description (or natural language) was superseded by the postulated use of symbolic notation systems, and current efforts are focused on obtaining objective measures that require the development of reliable coding systems (Donaghy, 1988) which, given that language is a code of a notational system (Krajcsi and Szabó, 2012), fulfill linguistic conditions: “The term ‘code’ as it is used here refers to the final result of three parallel processes, simplifying the original material, organizing it so that the relationship among its elements can be clear, and restructuring the whole for easy transmission.” (Dittmann, 1987, p. 39).

In the field of research into non-verbal communication any attempt to develop a movement alphabet must inevitably take into account Ray Birdwhistell’s Kinesic Notational System (KNS) (Birdwhistell, 1952, 1970) and the Facial Action Coding System (FACS), which is limited to facial expressions (Ekman and Friesen, 1978), without forgetting that there is a wide range of other approaches to this problem (Barker and Collins, 1970; Kendon, 1981, 1997; Crivelli et al., 2016).

In terms of notational systems it is important to highlight the following: (1) the most important aspect is not the tokens but the ability of the system to provide an exhaustive representation of people’s anatomical possibilities for movement; (2) how easy or hard a notational system is to use must be evaluated in terms of the relationship between clarity and precision; and (3) the acceptance and use of a notational system is determined by the consensus reached among researchers of the scientific community in question.

Hirsbrunner et al. (1987) reviewed the problem of movement transcription and argued that although the required efficiency of the coding language cannot be replaced by video technology for recording visual information (or audible information when analyzing a multimodal system of communication), neither is greater efficacy achieved by developing impressive notational schema to transform audiovisual information into data. Faced with such a situation the authors adopted the diagnosis and proposed solution of Frey and Pool (1976): “…current difficulties in movement description do not originate from the complexity of phenomena to be described, but from the investigators’ failure to base their coding systems on the principle of time-series notation.” (Hirsbrunner et al., 1987, p. 100).

The Bernese Time-Series Notation invokes a classic expression coined by Frey and Pool (1976), one which has proved highly powerful at resolving the continuity of the behavior stream (unifying speech and movement) by using nominal or categorical codes and seeking to detect space-time patterns. It starts by obtaining matrices of recorded data by transcribing movements, whether simple or complex, and thus generates a large amount of simultaneous data over time. Hirsbrunner et al. (1987) praise its possible application to the different parts of the body during movement and, after two decades of successive technological advances, the hurdle once posed by frame-by-frame transcription has now been totally overcome, thus ensuring high degrees of precision when obtaining data.

In this context, and having carefully reviewed the literature on the analysis of movement in the field of dance (Hutchinson-Guest, 1984) and physical appearance (Fink et al., 2015), as well as the most well-known and scientifically sound notational systems (Laban, 1926; Benesh and Benesh, 1956; Eshkol and Wachman, 1958; Eshkol, 1971; Bartenieff and Lewis, 1980; Farnell, 1996; Guest, 2011), we sought to develop a notational grammar of body movement which we call the Common Morphokinetic Alphabet (CMA).

The term “morphokinetic” is defined as a temporally demonstrable change in properties and spatial design of body motion form. By “common” we understand two things: (1) the notation system can be communicated and learned, as a balance is sought between clarity and precision and (2) the notation system shares the logic of meaning, physical identity/semantic content, which emerges from the writing of movement in the notational systems reviewed according to the choreographic model. Finally, the concept of “alphabet” denotes the conventional and discretional nature of the tokens and connotes the material condition sine qua non required to develop a notational system governed by grammatical rules.

In previous publications (Izquierdo and Anguera, 2001; Anguera and Izquierdo, 2006; Izquierdo, 2010) we have mapped out the different facets of this theoretical and methodological proposal that forms part of the movement observation process within the field of psychology (Chinellato et al., 2015; Castañer et al., 2016; Anguera et al., 2017). Now our aim is to present the CMA grammar and the criteria for its use in systematic observation studies. The following sections address the theoretical and methodological basis of the CMA notation, the grammatical formalization of the morphokinetic description, the general criteria for use in the coding format and as a free transcription system, and, finally, the possibilities offered by this notational system.

## Theoretical-Methodological Basis of the CMA

Advances in the study of non-verbal behavior and communication have led to the need for suitable systems for transcribing movement that are capable of incorporating continuous developments in visual and computer technology (Archer, 1991; Anguera, 2003; Blanco-Villaseñor et al., 2003; Portell et al., 2015a,b).

The range of possibilities offered by visual records and the physical analysis of behavior in the context of everyday human activity was clearly illustrated by the pioneering photographic work (in some cases, including magnificent images of reality) of cultural anthropologists, such as Mead and Bateson (1942) and Efron (1942), or clinical researchers, such as Scheflen (1964), who used images taken from film stills to indicate the path of gestures. Despite the promise of this early work, however, the relationship between visual records and the space-time analysis of movement in interactive and non-interactive situations has, as pointed out by Ekman (1964) faced a number of significant problems, some of which concern the KNS.

The warning raised in the context of interdisciplinary research into social behavior (Farnell, 1999) about the dangers of reducing kinesic descriptions to the anatomical functioning of the human body in order to achieve greater analytic rigor concerns the way of interpreting the application of kinesiology to kinesics more than it does the fact of basing the choreographic model of notation on anatomical and biomechanical knowledge (Shafir et al., 2016). In sum, “a comprehensive movement writing system has to resolve several technical difficulties. Human actions take place in three dimensions of space and one dimension of time and mobilize many parts of the body simultaneously. […]. The task is complex, surely, but not insurmountable […]” (Farnell, 1996, p. 868).

The CMA aims to code the visual form of body movement by describing it as a configuration sculpted in space-time. Each new configuration perceived by the observer implies a demonstrable change with respect to the immediately previous one. The change in configuration includes total or partial mobility of the body and relative stillness with respect to the following position, and, whenever necessary, the initial position can be maintained as a basic reference point for subsequent changes.

In terms of the spatial description of body movement a determining feature is that the body has a large number of degrees of freedom when executing movements. Bearing in mind this principle, CMA notation of spatial points is geared toward what is specific about the spatial design of a movement in accordance with the objective of the observation (Frey and Pool, 1976).

Given that body space is located in space/setting, CMA notation considers the movements through space that we can make with our body and the relationship between the use of space and overall body positions, that is, physical postures (standing, sitting, kneeling, lying down, etc.) and the postural movements produced by a form of established behavior (Mehrabian, 1969; Argyle, 1973; Poyatos, 1986).

In terms of timing, the morphokinetic description of a series of movements involves making a decision about the time interval to be used in order to obtain a good resolution of discontinuity, which results in the presence or absence of certain primary data that are considered in light of what is significant for the analysis (Page, 1996). Here a discretional criterion is used, which ranges from the frame-by-frame reading of the image to viewing at normal speed (Hirsbrunner et al., 1987).

A complete understanding of the temporal structure of movement phenomena involves the notation of the duration and temporal form (i.e., simultaneous or sequential) of movements. In addition to qualities concerning the speed, intensity and amplitude of changes, the use of signals derived from the physical appearance of the moving subjects and their socio-historical, cultural, and linguistic context are also transcribed in order to distinguish variations and individual differences in the kinesic form and style (Scheflen, 1972; Poyatos, 1986; Kendon, 1997).

From a methodological perspective the CMA has notable potential in that it is able to objectify behavioral units at the micro level due to the way it breaks up the stream of behavior (Condon and Ogston, 1970), and this gives it important analytic properties for subsequent empirical processing.

The first part of the analytic process consists of transforming the kinesic reality of human movement into units of behavior that are later turned into data with the aid of an observation instrument developed ad hoc; these data must be suitably managed before being analyzed, a task for which there are various approaches. Thus there are four stages that are necessary from a methodological point of view and that provide the CMA with its required consistency.

(1) The creation of molecular units (Schegloff, 2000) is a prior condition in the development of an observation instrument (Anguera and Izquierdo, 2006). In our view, demarcating the unit of behavior is clearly linked to the specific setting of objectives, and it must also be possible to demarcate, name, and define each unit.
(2) The decision taken regarding the demarcation of units opens the way to the development of an observation instrument: the field format. Originally, in the work of Weick (1968, 1985), this was a simple recording technique, but it has subsequently been revisited, developed (Anguera, 2003; Sánchez-Algarra and Anguera, 2013), and used widely in numerous fields, especially those involving movement, such as sport (Anguera et al., 2003). The development of the instrument involves the following steps: (a) Establishment of the criteria or axes of the instrument, which are set in accordance with the study objectives (for example, in observing a person who is learning to swim these might be area of the swimming pool, entering the water, submersion, equilibriums, displacements, etc.). Some of these criteria may be broken down hierarchically into others. (b) Listing of behaviors/situations (this list is neither closed nor exhaustive, and is known as the catalog) corresponding to each one of the criteria, and noted according to the information provided by the exploratory stage of the study. For example, starting from the criterion entering the water the list of behaviors could be entering feet first with help, entering from a sitting position on the edge of the pool without help, entering head first without help, etc. (the etc., indicates precisely that further behaviors can be added as the list is not closed). (c) Assignment of a decimal coding system to each one of the listed behaviors/situations that are derived from each one of the criteria. This means that any of the behaviors or situations can be displayed in a hierarchical system of lower order. Depending on the complexity of the case in question or the desired range of molecularity, these coding systems may be double, triple, etc. For example, the codes of the criteria would be 1 (area), 2 (entering the water), 3 (submersion), etc. And from 2 we could derive 2_1 (entering feet first with help), 2_2 (entering from a sitting position on the edge of the pool without help), 2_3 (entering head first without help), 2_4 (entering by jumping feet first from the side of the pool without help), etc. However, from 2_2 we could also derive 2_2_1, 2_2_2, 2_2_3, and so on successively^1^. (d) Drawing up of a list of criteria configurations. The configuration is the basic unit in recording field formats, and consists of linking together the codes corresponding to simultaneous or concurrent behaviors, thus enabling an exhaustive recording of the behavior stream and greatly facilitating the subsequent analysis of data^2^. For example, in the event that four criteria have been proposed:

1_3 2_4 3_2_1 4_2
1_3 2_3 3_2_1 4_2
1_2 2_3 3_2_4 4_4
…

(3) Having a tailor-made field format offers enormous flexibility in terms of data gathering, but it must be properly managed if the data in question are to be positioned in a way that optimizes their subsequent analysis. Given that the field format configurations are chains of simultaneous codes (synchronous relationship), and the sequence of these criteria configurations is established over time (diachronically), the modification of a single code over time is sufficient to yield the recording in the next row. Furthermore, the passage of time can be measured in conventional units (seconds) or in frames, and it is even possible to consider a conventional interval of any chronometric unit for each row of the matrix. In other words, the code matrices obtained will have, at most, the same number of columns as there are field format criteria, while the number of rows will depend on the successive changeability of the observed situation. This is perfectly in keeping with the proposal of Coster (2005, p. 17) as regards the preparation of data corresponding to postural dynamics, and is consistent with the structure of time-series notation. In this regard, there is a correspondence with the gathering of homogeneous data through the “restrictive coding” suggested by Frey and Pool (1976). Coster, following the way in which music is written, refers to the *horizontal* dimension, indicative of diachrony, and the *vertical* dimension, corresponding to synchrony or concurrence of behaviors. This proposal was found to fit perfectly with that resulting from the data management obtained when recording by means of field formats.
(4) Once the recorded data have been suitably managed a decision must be made as to the most appropriate analytic technique, always bearing in mind the objectives proposed in each case and the corresponding design. The three favored options, in light of their analytic potential, are lag sequential analysis (Sackett, 1980; Bakeman and Gottman, 1986; Bakeman and Quera, 1995), used to detect, if present, patterns or regularities in the series of recorded behaviors; detection of T-Patterns (Magnusson, 1996, 2000; Magnusson et al., 2015), which has a wide range of applications (Anolli et al., 2005), including non-verbal communication (Haynal-Reymond et al., 2005) and facial expressions (Merten and Schwab, 2005); polar coordinate analysis (Perea et al., 2012; Castañer et al., 2016), and time-series analysis on the basis of categorical variables (Bakeman and Gottman, 1986; Albert, 2001). Graphical representations of the temporal structure of body movement during a given period of time are also of great interest (Frey et al., 1982).

## Grammatical Framework of the CMA

Chomsky referred to generative grammar as “a system of rules that in some explicit and well-defined way assigns structural descriptions to sentences” (Chomsky, 1965, p. 8). The function of these rules is to specify whether the minimum terminal units of syntactic function comprise well-formed strings (phrase markers).

From the methodological point of view, Chomsky (1956) bases his investigation of the syntax of a natural language on the detailed analysis of what traditional grammar has to say about a simple statement. To this end he analyzes the following example: *sincerity may frighten the boy*. After considering the example from different perspectives he distinguishes three levels of information which may be extracted from the sentence. Each level implies referring to notions used in the syntactic and morphological analysis of the language; notions, such as “nominal phrase” and “verb”, from the first information level, are clearly distinguished from functional grammatical notions (e.g., subject, predicate, direct object, etc.) on the second level. The lexical and grammatical elements appear on the third level. Chomsky aims to determine “how information of this sort can be formally presented in a structural description and how such structural descriptions can be generated by a system of explicit rules” (Chomsky, 1965, p. 64).

**FIGURE 1:**
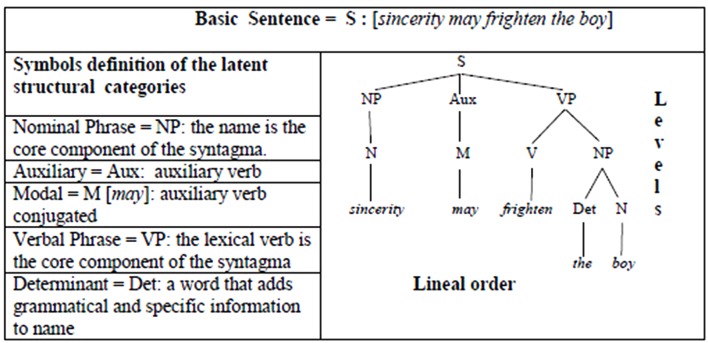
Triple structural information of the phrase marker: tree diagram.

In terms of our grammatical framework for the morphokinetic alphabet, it is sufficient to consider these two questions in relation to the first level of information: the breakdown of the sentence [S] into successive series on the basis of nominal [NP] and verb [VP] syntagms. The phrase marker indicates three types of information: (1) category labels (i.e., NP, V, Det, etc.); (2) the hierarchical arrangement of these categories; and (3) the linear order of the terminal string (**Figure 1**). The linear order of the terminal string from the category symbol ‘S’, which represents “Sentence”, is obtained by applying a sequence of rewriting rules (**Table 1**).

**Table 1:** Branch generation of the terminal string for a given grammar.

Example	

Given an initial string S	

Production Set P	Derivation
Rl. S→ AB	AB (Rl)
R2. A→ CD	CDB (R2)
R3. C→*c*	*c*DB (R3)
R4. *D*→*d*	*cdB* (R4)
R5. *B*→*b*	*cdb* (R5)

Terminal string *cdb*	

G = ({cdb}, {ABCD}, S, P)	


*Applying the sequence of production rules P the substitution of the element on the left of the rule by the one on the right is written on each derivation line. G is the grammar symbol, lower-case letters are the terminal symbols, and capital letters are the non-terminal symbols used by the generative grammar G.*

### Movement-Phrase Structure

By analogy to Chomsky’s procedure, the analysis of the structural components of a terminal string of the morphokinetic alphabet must answer three basic questions present within the movement notation systems reviewed (Izquierdo and Anguera, 2001; Izquierdo, 2010): “What has moved?” “What has changed?” and “How has it changed?” The information provided by Laban Notation, Benesh Movement Notation, and Eshkol-Wachman Notation in answer to these basic questions differs slightly as they have different reference frameworks and orthography. The Laban and Benesh systems have a richer vocabulary than the Eshkol-Wachman system when it comes to describing movement (form, space, time, and temporalization) and the qualitative aspects or “how one move” (Neagle et al., 2002; Guest, 2011).

Within the framework of the CMA, the first question requires us to name and identify the bodily form of movement: part of the body + figure. The second question must be answered by specifying spatial and temporal references with respect to position (overall physical posture, position on the floor, or any other aspect related to the maintenance of overall physical posture), orientation (position in the movement plane, direction, and height), and the duration of movements and their structure in time. The final question (How has it changed?) involves identifying the contextual factors which may affect the form of movement and classifying the specific mode of the motor action. The contextual factors, considered as invariant at least within the same observation session, include the situation where the activity takes place, the baseline body and psychosocial conditions of the person (or persons) in movement, the reference culture, and the acquired habituation in executing the movements (i.e., slow movers, lively movers, etc.). The qualities perceived for specific movements, that is, the impression we form of speed (e.g., slow/fast), intensity (e.g., gentle/strong), and amplitude (e.g., narrow/wide), as well as the use of physical appearance, including styles related to culture or social status that are not selected by the situation, are the aspects that classify the idiosyncratic differences observed in the execution of specific movements.

**FIGURE 2:**
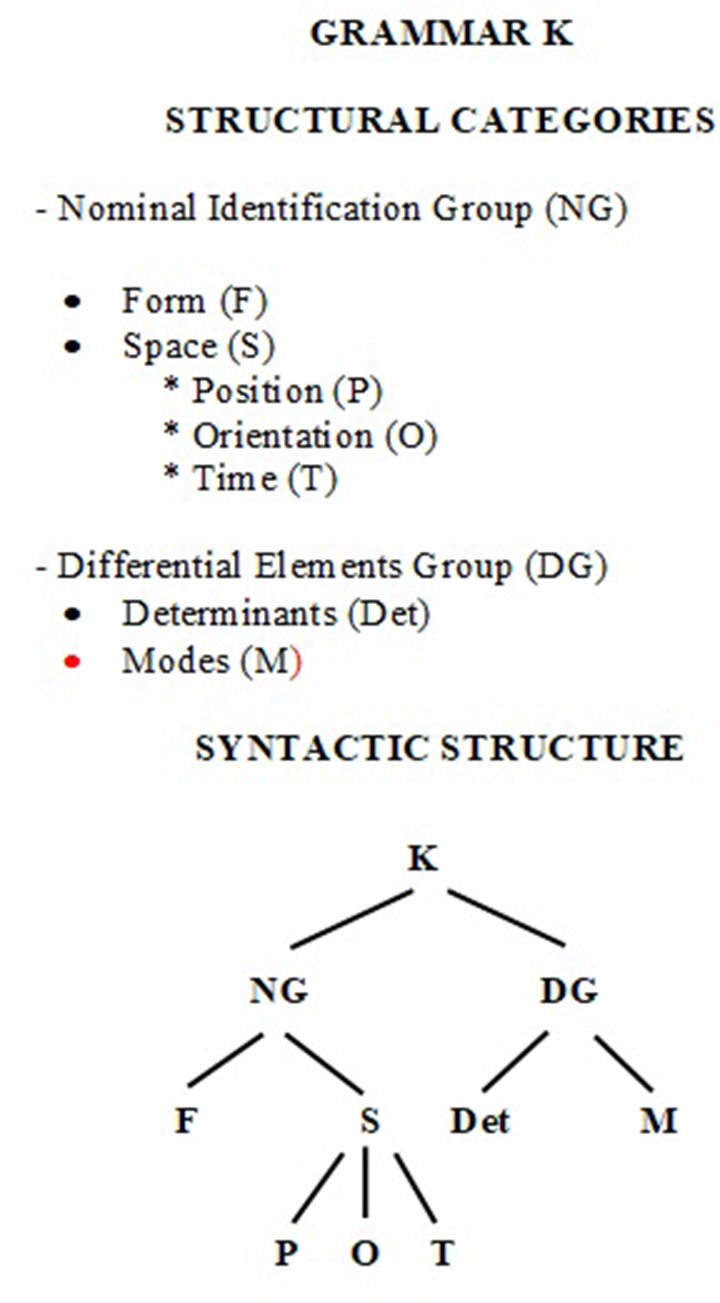
Syntactic components of the morphokinetic grammar K.

Insofar as the aim of the morphokinetic alphabet is to symbolize elements of the visual image that have been recognized, named, and labeled using the words/concept that represent body movement (e.g., up/down of shoulder), then the structured organization of the symbols of a morphokinetic alphabet may use, as one option among other possible ones, the same way of representing structural information as phrase marker grammar. Therefore, the information recognized, named, and labeled leads us, on the one hand, to the structural categories of the morphokinetic expressions and, on the other, to the morphokinetic categories established in each case for the coding protocol. At the structural level, the nominal component is referred to by the symbol NG (meaning “Nominal Identification Group”) and the labeling component is referred to by the symbol DG (meaning “Differential Elements Group”). The breakdown of the components of these structural categories into hierarchical levels is shown in **Figure 2**.

Let us analyze a visual description with words, chosen from among the many possibilities to be found in common written texts or works of literature, and apply the proposed structural categories. The chosen text (from Poyatos, 1994, p. 140) is “*Every morning* [my father] *attended mass*, [all the time^∗^] *with both knees on the floor, his hands together, pointing upwards at chest level, his hat on top of them*” (Alemán, GA, I, I)^3^, and thus we obtain:

‘*Every morning*’ > DG: it is context [Det]: temporal reference;
‘[my father]’ > DG: it is context [Det]: personal reference with a social basis: family relationship;
‘*attended mass*’ > DG: it is context [Det]: religious activity: selects the repertoire of action;
‘[all the time^∗^]’ > reader’s inference > NG: it is time [T]: duration of the whole body figure;
‘*with both knees*’ = [kneeling] > NG: it is form [F]: body part + figure;
‘[kneeling] *on the floor*’ > NG: it is position [P]: location in physical space;
‘*his hands together*’ > NG: it is form [F]: part of the body + figure;
‘*pointing upwards at chest level*’ > NG: it is orientation [O]: vertical axis, sagittal plane;
‘*his hat on top of them*’ > NG: it is form [F]: supporting object;
‘(…) *on top* (…)’ > NG: it is orientation [O]: height scale;
‘*his hat on top of them’* DG > it is modal [M]: it is form: familiar/strange emphasis.

This exercise is merely an initial approach to the adaptation of structural symbols to the morphokinetic information expressed in a word or group of words.

### Rewriting Rules

Continuing with the “*phrase-structure*” analogy, let us consider a simple example of syncopated verbal-morphokinetic description (the order follows the conventional above-cited written text): “*every morning, attended mass, all the time, kneeling, hands together, pointing upwards chest, and hat on top hands*” (1). (The commas separate the word symbols; note that there are symbols composed of several words).

Representation of (1) using labeled square brackets (K is the initial symbol. *Vid* **Figure 2**):

[NG[F[hands together]F SK[P[kneeling]P O[pointing upwards chest]O T[all]T]

$$S]NG\,\;DG{[Det}^{\left[\text{every morning, attended mass}\right]}{\text{Det M}}^{\left[\text{hat on top hands}\right]}M]DG{]}_{K}$$

Assuming that this formalizatiwic entities, the branch rewriting rules of the grammar K are:

R1. K → NG DG
R2. DG → Det M
R3. NG → F S
R4. S → P O T
R5. F →*hands together*
R6. P →*kneeling*
R7. O →*pointing upwards chest*
R8. T →*all the time*
R9. Det →*every morning, attended mass*
R10. M →*hat top hands*

The “base mold” of grammar K is acceptable within the restrictive framework imposed by our interpretation of the structural components of the morphokinetic description. In this regard, the formalization of the systematization carried out here is characterized by the negligible abstraction of the categorical notions, and in concert a clear application effect on the grammatical ordering of the symbolized morphokinetic expressions. In some ways, the analogical attitude (as if) indicates that we have defined an intermediate space between the branching rules of phrase marker grammar and the rules of action.

## Criteria for Use of the CMA

The proposed formal method for determining the hidden structure of “natural” morphokinetic expressions provides a syntax that orders the symbols of the morphokinetic alphabet: F ∩ S (P, O, T) ∩ Det ∩ M. As we have just seen, each element of the terminal string is a member of K in NG ∩ DG. For example, “*smoothly*” is a member of K in DG ∩ M.

The formalized syntax of the morphokinetic phrase serves as a guide not only when the movement image is observed live or through the viewing of photographs, film, or video but also when working with written texts. The grammar K channels the search for answers, and their writing, to the three basic questions: “What has moved?” “What has changed?” and “How has it changed?”

One way of optimizing the structural categories is to link them to the movement behavior criteria established in the field formats. The folder of each structural category can be displayed in as many sub-folders as necessary. Each folder contains complementary or alternative codes and, in addition, there are open options and specific catalogs (in accordance with the morphokinetic protocol created) so that the observer/analyst of movement selects, for each recording level, the codes that describe the image of the observed movement. This procedure can be carried out relatively easily using a database, such as Access.

In the example “learning to swim” (see above), one of the recording axes established in the field format is the criterion *entering the water* (code 2). Let us suppose that code 2_2, *entering from a sitting position on the edge of the pool without assistance*, requires a simplified morphokinetic description for some reason. In this case, the file of code 2_2 would contain the sub-files F, S, Det, and M, and the coding dimensions considered to be of interest, the codes, and the stipulated measurement specifications would all be displayed for each one of these sub-files. We propose two examples (**Tables 2**, **3**) of simplified morphokinetic coding [K]. See the list of symbols in **Table 4**.

**Table 2:** Example 1: CMA codes.

Code 2_2: Entering from a sitting position on the edge of the pool without help. morphokinetic analysis of the shape of the state of the torso
F	S			Det	M
			
	P	O	T		
41/cox	1	2	7.00	1	42

Description					

Standing up (1^P^) + convex (411^F^) + forward (21f^O^) + duration (7^T^) + first attempt (1^Det^) + tense (4^M^) + slow, deliberate waiting time (2^M^)					

Morphokinetic syntactic configuration Code 2_2 1-K: 1 411 21f 7 1 4 2					


**Table 3:** Example 2: CMA codes.

Code 2_2: Entering from a sitting position on the edge of the pool without help. Analysis of the sequence of positions of large head movements with or without speech
F	S			Det	M
			
	P	O	T		
111/lab	1	31	7.00	1	29

Description					

Standing up (1^P^) + labile head (111^F^) + rotation speech (31^O^) + duration (7^T^) + first attempt (1^Det^) + slow, deliberate waiting time (2^M^) + speech fear (9^M^)					

Morphokinetic syntactic configuration Code 2_2 2-K: 1 111 31 7 1 2 9					


**Table 4:** CMA selection of symbols (#) for free transcription.

Form	#	Spatial Points	#	Other Notations	#
**Body parts**		**Location**		**Body side**	
*Joint/areas*		At the center	000	Right/left	F′/F,
Neck	1	At center front	100	**Body participation**	
Shoulder	2	…	…	Bilateral movement	F^∗^
Elbow	3	**Physical body posture**		Unilateral movement	F
Knee	4	Standing up	1	**Form timing movement**	
…	…	Sitting	2	Simultaneous compound	^..^F
Head pole	11	…	…		
	12				
	…				
Arms pole	21	**Orientation**		Sequential compound	F
	…				
(e.g., index fingers)	(232)				
Legs pole	31	Vertical plane	11	**Height scale**	
	32	position	12	**body/vertical axis**	
	…		…		
Trunk	41	Sagittal plane	21	Higher…….Lower	x5,…,x1
	42	position	22		
	…		…		
**Figure**		Horizontal plane	31	**Time-duration**	
		position	32		
			…		
Drawing and poetry	abbr./words	**Direction**		Non-recorded duration < 1 s	<
Technical terms	abbr./words/numbers	High/low V axis	h/w	≥1 s (decimal notation)	1.00
(e.g., labile head)	(111)	Right/left H axis	r/l	Minutes (u:v)	1:00
(e.g., convex torso)…	(411)…	Forward/backward S axis	f/k	**Determinants and attributes**	abbr./words/numbers
				(e.g., slow)	(2)
				(e.g., tense)	(4)
				(e.g., speech fear)	(9)
					…
		Oblique directions	rf…	**Transcription number**	n-K


*The readers should read these column by column.*

Example 1: Analysis of the shape of the torso at the current moment in code 2_2 (**Table 2**).

Example 2: Analysis of the sequence of positions of large head movements with or without speech in code 2_2. See **Table 3**.

When the aim is to prepare the simplified schema for the data collection work that will subsequently be carried out, the CMA functions as a free transcription system. In the context, it is necessary to economically transcribe the movement action for their analysis (**Table 4**). Free transcription also converts the kinesics present in written natural language into movement scores.

One example is the compound and sequential gesture described by Kendon (1987, p. 85) – “[action context and speech:…]. In this gesture he placed his two extended index fingers side by side and then extended both arms away from himself and upwards in the direction of the door”. It is transcribed as follows (**Table 5**):

**Table 5:** Transcription from Kendon example.

	Identifiers
1-K [transcription number]	
Action context […^*DG*^]	
Action sign [indicating to leave: Fext]	
Speech [“…, so maybe we ought + GESTURE”]	
Sequence actions order F	Movement behavior with CMA
1	F^∗^232ext 11 [“two^F∗^ extended^Fext^ index fingers^F232^ side by side^V11^”]
2	F^∗^21ext 21fdoor [“then extended^Fext^ both^F∗^ arms^F21^ away from himself and upwards ^O21h^ in the direction of the door^Detdoor^”]

Morphokinetic syntactic configuration 1-K: F^∗^ 232ext_21ext 21h door	


Finally, the use of general scripts (e.g., Labanotation) is compatible with our grammar. Any of these transcriptions can be converted to a decimal coding system or translated to the CMA vocabulary (alphanumeric and word symbols), achieving an accurate reproduction of the motor action, without loss of meaning.

## Conclusion

The process of observing human movement depends on how morphokinetic changes are perceived and described. The CMA notation system simplifies, organizes, and restructures (Dittmann, 1987) the morphokinetic changes in the psychological space of the observer/analyst as distinct descriptive phrases or movement configurations. Changes in the body figure are demarcated by the variables of space and time, and the identification is completed through the inclusion of words that mark the context of activity and classify the movement’s linguistic space.

Grammatical formalization is a way of forming acceptable symbol strings in accordance with the properties assigned to the syntactic component. The grammar has been developed here on the basis of phrase marker grammar. The simplest movement phrase, regardless of the size of the morphokinetic unit being considered, must be able to be analyzed as a basic expression: the visual form of the movement described provides information about, on the one hand, the perceived constraints between body, space, and time, and, on the other, the perceived connection between body, space, and time and the particular execution of the motor action.

Without doubt the most delicate question related to precision concerns the selection of movements and their description with respect to the reference framework adopted: body parts/space-time and other attributes.

Finally, the CMA may be useful for several basic reasons set out in this article: (1) it gives structure to the processes of identifying, writing, reading, rebuilding, reflecting upon, and analyzing raw data in the form of time function (video record); (2) it offers an open and flexible coding format that is compatible with the solutions offered by other notational systems for transcribing body movement; (3) it meets the frequent need to combine molar and molecular units in the same recording as if it were a zoom, in other words, without losing the unitary view of the whole body under consideration; (4) it allows the computerized management of visual notations; (5) it combines the principles of synchrony and diachrony of movement behavior, which enables advanced analytic techniques (time-series, sequential analysis, T-pattern analysis, etc.) to be applied to the matrix of reliable data; and (6) it performs an appreciable function as a transcription system in situations involving direct observation and when working with the kinesics of written texts.

The CMA is designed as a basic framework for developing specific coding schemes of body movement in a social context. In this regard, the next step will be to build a guide for recording and coding movement behaviors in each area of study. Further research is required on the applications of the CMA in order to assess its potential and scope. It is expected that new initiatives will provide additional evidence about the versatility of the system and assurances regarding the reliability of the trials carried out by different researchers who are committed to the grammatical principles of the morphokinetic observation on which the CMA is based: simplified coding, field format time recording, and syntactic rules of descriptions.

## Author Contributions

CI developed the project ‘Common Morphokinetic Alphabet’ (CMA) under supervision of MTA. Both authors reviewed the literature and discussed the references included in the proposal of a grammatical framework of body movement for observing and coding morphokinetic records in systematic observation studies. MTA incorporated the four stages that structure the process of systematic observation. CI designed the path followed in the conceptual analysis of the problem of movement notation, and made the draft of the manuscript. This manuscript is result of shared work.

## Conflict of Interest Statement

The authors declare that the research was conducted in the absence of any commercial or financial relationships that could be construed as a potential conflict of interest.

## Footnotes

1 *Lower-case hyphen*: links the digits that form a new code comprised by two or more already-assigned codes.

2 *Empty space in the code chain (rows)*: indicates the concurrence of different codes.

3 Authors’ own translation. The original Spanish text is: “*Cada mañana* [mi padre] *oía su misa*, [todo el tiempo^∗^] *sentadas ambas rodillas en el suelo, juntas las manos, levantadas del pecho arriba, el sombrero encima de ellas* (taken from *Guzmán de Alfarache* by Mateo Alemán: see Poyatos, 1994, p. 140).
